# The BUSCOPAN study: a randomized-controlled non-inferiority trial of a continuous butylscopolamine infusion versus placebo in patients with a renal colic not responding to oral non-steroidal anti-inflammatory drugs

**DOI:** 10.1007/s00345-020-03460-0

**Published:** 2020-09-19

**Authors:** S. Weltings, K. T. Buddingh, D. C. van Diepen, R. C. M. Pelger, H. Putter, M. Rad, B. M. A. Schout, H. Roshani

**Affiliations:** 1 Haga Teaching Hospital, The Hague, The Netherlands; 2 Erasmus MC, Rotterdam, The Netherlands; 3 LUMC, Leiden, The Netherlands; 4 Alrijne Health Group, Leiderdorp, The Netherlands

**Keywords:** Urolithiasis, Ureteral calculi, Renal colic, Butylscopolammonium bromide

## Abstract

**Purpose:**

To investigate whether placebo is non-inferior to continuous infusion of butylscopolamine in patients with renal colic*.*

**Methods:**

We conducted a placebo-controlled, multicenter, double-blind randomized clinical trial (RCT) including 128 patients with renal colic (confirmed by ultrasound or CT-scan). Patients were randomized to receive either continuous IV butylscopolamine 100 mg/24 h or placebo (saline). Primary outcome is the amount of opioid escape medication used, measured in doses administered. Secondary outcomes are pain measured on a Numeric Rating Scale (NRS), side effects, and time of drug administration. Non-inferiority was assessed using linear regression with robust standard errors, with non-inferiority limit set at 0.5 units of escape medication.

**Results:**

Median number of doses of escape medication was one in both groups. The number of extra doses in the placebo group compared with the butylscopolamine group was 0.05, with a 95% robust confidence interval (CI) of 0.38–0.47. Upper limit of the CI remained below the non-inferiority limit of 0.5 (*p* = 0.04). No differences in secondary endpoints were seen between the groups.

**Conclusion:**

Placebo is non-inferior to continuous IV butylscopolamine for pain relief in patients with renal colic. Based on this study and previous evidence, there is no role for continuous butylscopolamine IV in the treatment of renal colic.

Trial NL7819

## Introduction

A renal colic, mostly produced by a calculus in the upper urinary tract, is one of the most severe forms of pain known. Non-steroidal anti-inflammatory drugs (NSAIDs) are the agents of first choice to control the pain in these patients. If NSAIDs are insufficient or contra-indicated, titrated intravenous (IV) or intramuscular opioids are generally recommended as a second step [1, 2]. Additionally, anticholinergic spasmolytic drugs have been prescribed to patients with renal colic since the nineteenth century [3]. The rationale is that such drugs may induce smooth-muscle relaxation by inhibition of the action of acetylcholine on the muscarinic receptors in the wall of the ureter. One type of antimuscarinic that received interest over the past decades is butylscopolamine.

In the Netherlands, continuous IV infusion of butylscopolamine has long been used for pain control in patients hospitalized for renal colic. It is advised by the Dutch National Guideline on kidney stones to consider as a second step after administering NSAIDs, and before opioids [4]. IV butylscopolamine is an integral part of renal colic management in The Netherlands, and we estimate that it is administered to several thousands of patients per year. Its use in other countries varies between institutions; there are no data on how widespread this practice is.

The use of butylscopolamine stems from tradition, not based on scientific evidence [3]. Previous studies dismissing buscopan as ineffective have noteworthy flaws. A handful of studies failed to prove effect of *oral* butylscopolamine on pain in renal colic [3, 5–8]. A few trials assessed efficacy of IV butylscopolamine with a single dose of 20 mg [9]. No benefit of butylscopolamine in reducing opioid or metamizole need in renal colic was seen. Because of the rapidly declining plasma concentration of butylscopolamine, a single dose might not suffice for a therapeutic effect [9]. One recent RCT studied the effect of 80 mg IV butylscopolamine compared to placebo on renal colic and concluded that there was no statistical difference between the placebo and butylscopolamine group [10]. However, this study was poorly suited to detect a beneficial effect of butylscopolamine, because patients in both groups received an initial dose of butylscopolamine.

In 2016, the uncertain benefit of a continuous butylscopolamine infusion was identified as an important clinical knowledge gap by the Dutch Association of Urology, supported by the Netherlands Patients Federation [4, 11].

We conducted a multicenter double-blind randomized-controlled trial (RCT). The primary objective was to assess whether placebo is non-inferior to continuous intravenous infusion with butylscopolamine in patients admitted for pain due to renal colic with regards to amount of opioid escape medication needed.

## Materials and methods

This randomized-controlled clinical trial recruited patients in two general hospitals in The Netherlands between January 2018 and November 2019. The trial was registered in the Netherlands Trial Registry (Trial NL7819). The protocol was approved by the independent ethics committee (IEC, approval number 17-081) and participants gave written informed consent prior to inclusion. The study has been conducted following the Guideline for Good Clinical Practice.

### Patients

Eligible patients were adults presenting with a renal colic, when pain was not under control with oral NSAIDs, they were admitted to the urological ward for analgesics. Confirmation of a renal calculus by ultrasound or CT-scan was required for inclusion. Exclusion criteria were pregnancy or lactation, contra-indication or known allergy to any of the drugs used (NSAIDs, morphine, paracetamol), temperature > 38.5 °C in the 24 h before inclusion or receiving antibiotics for urinary tract infection, or indication for immediate drainage of the upper urinary tract.

The primary outcome in this study was the amount of escape medication used during the 24-h period of observation, measured in doses administered. Secondary endpoints were reduction in pain, measured using Numeric Rating Scale (NRS), time until last need for a dose of escape medication, side effects, use of anti-emetics, and surgical interventions necessary for ongoing pain.

### Randomization and blinding

Patients were randomized in a 1:1 ratio to one of two study-arms using sequentially numbered opaque sealed envelopes. No stratification was done. Randomization of treatment was determined in advance using a random numbers table. Patients, clinical staff, and investigators were blinded to the allocation. Study allocation remained blinded until completion of the entire study.

### Study procedures

All patients were given 1000 mg oral paracetamol four times daily and 50 mg oral diclofenac three times daily. They also received oral tamsulosin 0.4 mg once daily. Escape analgesics consisted of piritramide 15 mg subcutaneously as needed up to a maximum of five times. Furthermore, an IV anti-emetic was prescribed as needed. In one arm, patients received butylscopolamine (Buscopan®, Sanofi SA) 100 mg/24 h via an intravenous continuous infusion, and in the control arm, saline was given as a placebo.

Patients were asked to rate their level of pain using NRS at the start of the study period and subsequently at 1, 4, 8, and 24 h. Also, experience of side effects was asked. Escape medication was used to maintain adequate pain relief, and a pain score below 4 was accepted as adequate pain management. After 24 h, the study period ended and patients received standard care from there onwards.

### Sample size and statistical analysis

The sample size was calculated using a one-sided, two-sample t test [PASS version 08.0.16 (Hintze J, 2008); NCSS, LLC, Kaysville, Utah, USA].

The null hypothesis assumed that placebo is not inferior to treatment with butylscopolamine on the effect of using extra analgesics. We deemed a non-inferiority margin of 0.5 as clinically relevant. With the power fixed to 80% and a one-sided significance of *α* = 5%, the required sample size to detect non-inferiority was calculated to be 51 patients in each arm. The data are drawn from populations with standard deviations of 1.0 and 1.0. Allowing for 20% drop-out, inclusion of 128 patients was planned.

All statistical analyses were performed using SPSS statistics, version 26.0 (IBM, Armonk, NY, USA). After exclusion of patients that did not meet inclusion criteria, all analyses were performed on an intention-to-treat basis. Normality of data was analyzed using the Shapiro–Wilk test. Data with skewed distribution were analyzed using non-parametric tests, with exception of the primary endpoint. Since the primary endpoint is analyzed in the context of a non-inferiority study, analysis of the primary endpoint was performed using linear regression with robust standard errors, to protect against non-normality of the outcome. The 95% confidence interval (CI) of the difference between the randomized treatment groups is based on these robust standard errors. For the other endpoints, numerical continuous data were analyzed using the independent *t* test (and the Mann–Whitney *U* test for data showing skewed distribution). A *p* value < 0.05 was considered statistically significant.

## Results

During the study period, a total of 290 patients were admitted for renal colic in the two participating centers. After assessing for eligibility and exclusion criteria, 128 were randomized and data of 124 patients were available for analysis. Three of the four patients that were excluded after randomization developed fever soon after admission and required urgent upper tract drainage. The fourth patient withdrew for personal reasons. There were a few minor protocol violations: administration of morphine instead of piritramide in two cases and refusal of diclofenac in eight cases, and these were evenly spread among the two arms.

Sixty-two patients remained in each arm. Groups were not different when comparing for baseline characteristics, stone location, or stone size, as shown in Table 1.

**Table 1:** Baseline characteristics

	Buscopan (*n* = 62)	Placebo (*n* = 62)
Gender, *n* (%) Male	44 (71%)	41 (66%)
Female	18 (29%)	21 (34%)
Age, mean ± SD	49 ± 14	45 ± 15
BMI, median (range)	28.2 (18.8–42.5)	26.6 (20.0–47.0)
Creatinine at admission in mmol/L, median (range)	91 (49–180)	90 (53–169)
Stone side, *n* (%) Left	34 (55%)	38 (61%)
Right	28 (45%)	24 (39%)
Stone size in mm, median (range)	5 (2–20)	5 (2–18)
Stone location* *n* (%) Proximal	24 (39%)	22 (37%)
Distal	38 (61%)	39 (63%)
Hydronephrosis, *n* (%) None	8 (13%)	9 (15%)
Mild (grade 1–2)	47 (76%)	44 (72%)
Severe (grade 3–4)	7 (11%)	8 (13%)
Primary diagnostics, *n* (%) Ultrasound	42 (68%)	37 (60%)
CT	53 (86%)	55 (89%)
NRS score, mean ± SD At start of study	4.7 ± 2.7	4.7 ± 2.8

*Proximal; cranial of crossing ureter and iliac vessels. Distal; caudal of crossing ureter and iliac vessels

## Primary outcome

A total of 68 patients (55%) required opioid escape medication. The number of doses of escape medication required was 1.0 (95% CI 0.7–1.4) in the placebo group and 1.0 (95% CI 0.7–1.3) in the buscopan group. The number of extra doses of escape medication needed in the placebo group compared to the buscopan group was 0.05 (95% robust CI 0.38–0.47). The upper limit of the confidence interval remained below the non-inferiority margin of 0.5 (*p* = 0.04). This indicates that placebo is non-inferior to butylscopolamine.

### Secondary outcomes

**Fig. 1:**
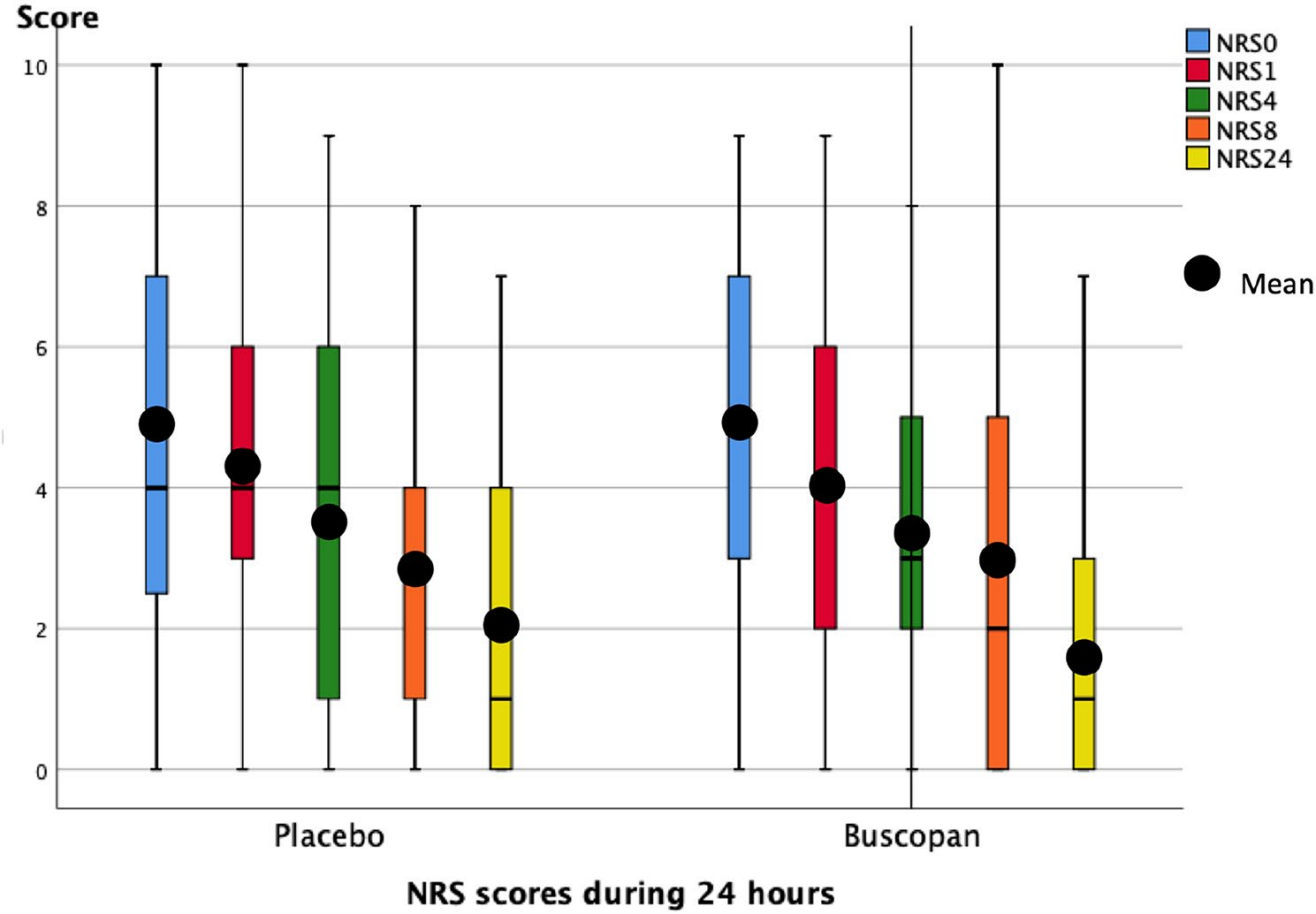
NRS scores during study period

Both groups showed a similar decrease in pain measured by NRS over time (Fig. 1). There was no statistical difference in time until last escape medication: a median of 7.0 h in the butylscopolamine arm and 9.3 h in the placebo arm. Side effects were reported by 24 patients with no statistical differences between the groups. There was no difference in amount of anti-emetics used and there were no surgical interventions for ongoing pain during the study period. Seventy-two patients left the hospital after the study period of 24 h to be further monitored in the outpatient department. The other 56 patients were given either standard care with analgesics or intervention, e.g., double J catheter, nephrostomy, or ureteroscopy.

### Follow up

The mean follow-up was 68 days in the butylscopolamine group and 60 days in the placebo group. More than 90% of the patients were stone free at approximately 2 month follow-up, of whom just under half had had a surgical intervention or ESWL in the meanwhile.

## Discussion

Pain caused by ureteral obstruction is related to increased tension in the walls of the renal pelvis on release of prostaglandins. Symptoms can be worsened by edema or inflammation in the ureter [3]. NSAIDs inhibit prostaglandin synthetase, which results in suppression of pain sensation and inflammation [7]. NSAIDs have already shown to be superior to opioids and paracetamol for the relief of pain in patients with a renal colic, resulting in less vomiting and less need for rescue analgesia [12]. Hyperperistalsis of the ureter, modulated by alpha- and beta-adrenergic receptors, plays a part in this physiologic process as well. Alpha adrenergic receptor antagonists provide smooth-muscle relaxation of the urinary tract, facilitating stone passage, but an analgesic effect has not been proven [13]. Antimuscarinic agents such as butylscopolamine can induce smooth-muscle relaxation and decrease of ureteral spasm as well [14]. Their effect on the gastrointestinal and biliary tract has been widely studied and accepted, but an analgesic effect in renal colic has neither been proven [15, 16].

A fair number of previous studies have been performed using different administrations of butylscopolamine, e.g., oral, intramuscular, or, in few cases, intravenously [3, 10]. Mostly single dose was used in these trials and mainly butylscopolamine was used as an additive in comparing different types of analgesics in patients with colic pain such as NSAIDs or opioids. A recent cochrane analysis showed that addition of antimuscarinics to NSAIDs is not superior to NSAID monotherapy regarding pain reduction and use of escape medication [17, 18].

All previous studies with *oral* butylscopolamine showed no benefit of this drug on renal colic [5]. This is hardly surprising, since oral administration results in a poor resorption of butylscopolamine of approximately 8% [16, 19]. Any significant effect of butylscopolamine is, therefore, only to be expected after parenteral administration. After intravenous administration, plasma concentration of butylscopolamine declines rapidly and the elimination half-life ranges between 1 and 5 h. The total clearance is 1.2 L/min, of which 50% is excreted as unchanged drug through the kidneys [9]. The pharmacological effect of a single dose completely wears off after 30–40 min [9]. A continuous intravenous drip instead of a bolus may, therefore, be given to maintain a pharmacotherapeutic effect.

Three studies were performed with a single dose of intravenous butylscopolamine. One RCT of Holdgate concluded that there is no support for the addition of butylscopolamine IV to reduce the need for opioids. However, this research studied the effect of a single dose of 20 mg butylscopolamine IV in patients suspected of renal colic, not confirmed in 40 cases [5]. One single blind study of Stankov et al. in 1994 showed a pain intensity reduction at 30 and 50 min after a 20 mg bolus of butylscopolamine similar to tramadol (100 mg IV), but significantly lower than values reached with metamizole (2.5 g IV) [6]. A third study compared a single dose of butylscopolamine (20 mg IV) with papaverine (60 mg IV) and pethidine (50 mg IV), and concluded that VAS scores were significantly higher in the butylscopolamine group [20].

Only one group studied the effect of continuous infusion of butylscopolamine (80 mg IV) on renal colic, comparing it with a placebo and a tramadol arm. They concluded a tramadol drip alone is a safe alternative for infusion with antispasmodic drugs. This study, however, had limited power to detect a benefit of butylscopolamine, because patients in both groups received an initial single dose of butylscopolamine [10]. Furthermore, the medical staff was not blinded to the study medication.

Our study was adequately powered and double blinded. All patients had confirmed renal stones on CT-scan (88%) or ultrasound. The study recruited a remarkably high proportion of all eligible patients and accrual was completed well within the planned time-frame of 24 months. The drop-out rate was low at 3%. It is, therefore, reasonable to assume that the study population accurately reflects the entire population of patients with renal colic and the findings may be extrapolated as such.

The study has a few potential limitations. In some hospitals, 120 mg of butylscopolamine per 24 h is used, rather than the 100 mg in this study. It is unlikely, but cannot be completely excluded, that the additional 20 mg would have changed the outcome of this study. A further limitation are the few minor protocol violations as described in the Results section. Eight patients refused diclofenac, but did receive piritramide when a renal colic arose.

On first thought, one might not expect a great impact of no longer administering continuous butylscopolamine for renal colic, considering that the drug is very cheap and has a favorable side effect profile. However, the preparation and monitoring of a continuous infusion does form a burden on the nursing staff. The patient is far more mobile without a continuous drug pump. Although butylscopolamine is generally safe, a recent update in the UK emphasizes potential dangers of this drug in patients with underlying cardiac disease [21]. Finally, it is common practice to advise the patient to stay hospitalized for several hours after stopping the infusion to evaluate if he or she remains pain free without butylscopolamine. Eliminating butylscopolamine from the treatment of renal colic could, therefore, shorten admission times and relieve pressure on emergency care beds.

## Conclusions

Placebo is non-inferior to intravenous butylscopolamine for pain relief in patients with renal colic. This adequately powered and double blinded trial confirms what previous limited studies already suggested: there is no benefit of intravenous butylscopolamine for renal colic. In our opinion, no additional studies are necessary for application of butylscopolamine for this indication. Patients experiencing renal colic should be treated with adequate analgesics (paracetamol, NSAIDs, and opioids if necessary) and there is no role for a continuous administration of IV butylscopolamine.

## Data Availability

All original data are available in Haga teaching hospital.
